# ‘There was no great ceremony’: patient narratives and the diagnostic encounter in the context of Parkinson's

**DOI:** 10.1136/medhum-2016-011054

**Published:** 2016-11-07

**Authors:** Jane Peek

**Keywords:** Neurology, Social science

## Abstract

This paper draws on stories of diagnosis that emerged from a broader narrative study exploring the lived experience of Parkinson's (n.37). Despite the life-changing nature of their diagnosis, participants' narratives highlighted considerable shortcomings in the way in which their diagnostic encounter was handled, echoing the findings of previous research in which it has been noted that ‘the human significance’ of diagnosis was passed over. Building on the literature, this paper provides empirical material that reveals the sensitivities involved at the moment of diagnosis. By examining both the structure and content of participants' narratives, this article discusses the diagnostic encounter in relation to three key concepts that connected many participants' stories: a ‘bareness’ or lack of ‘ceremony’, a sense of emotional and physical ‘abandonment’ and the impact on a person's illness story when faced with a ‘hierarchy’ of illness. This paper aims to raise awareness of contemporary issues related to the diagnosis of Parkinson's, and invites reflection on how diagnosis might be undertaken in a way that truly acknowledges its human significance.

## Introduction and background

Diagnosis is both a label and a process, the power of which lies in its ability to transform apparently random symptoms into an ‘organised illness’^i^ and to ‘hail’ people with a new identity of ‘patient-with-a-diagnosis’.^1^ To be ‘hailed’ by a diagnosis is usually ‘transformative’,^2^ a turning-point that ‘marks a day when life changes’^3^ and divides a person's life into a ‘before’ and ‘after’, a division that is thereafter ‘superimposed onto every rewrite of the individual's life story’.^4^ The moment of diagnosis is, of course, only a part of any diagnosis story, and yet it is widely accepted that the *manner* in which a diagnosis is delivered affects the immediate emotional response of the recipient as well as how they adjust and adapt to their new identity.^5^ And yet, during the course of a qualitative study exploring the lived experience of Parkinson's,^ii^ participants' narratives revealed considerable shortcomings in the manner in which the diagnosis encounter was handled. This is of particular concern given the deceptive nature of Parkinson's. Traditionally viewed as a neurodegenerative ‘movement disorder’, recent years have witnessed attempts to ‘dispel the myth that Parkinson's is benign’^iii^ and redefine it as a neurocognitive-psychiatric disorder involving multi-centric neurodegeneration that affects all aspects of everyday life.^6–9^ Nevertheless, it is still diagnosed clinically on the basis of ‘characteristic motor features’ (bradykinesia, resting tremor, rigidity or postural instability) and the lack of a straightforward diagnostic test means that the life ‘before’ might involve months, even years, of ‘diagnostic limbo’^10^ or even *mis*diagnosis. Given the progressive, degenerative nature of Parkinson's, most research exploring the patient perspective has focused on its impact on people's lives *after* diagnosis, exploring themes of unpredictability, uncertainty, loss of control, stigma, social withdrawal and social isolation^29^ ^11–16^ or examining issues relating to particular ‘patient groups’ in order to inform practice (eg, gender,^17^ ^18^ age or ‘stage’ of disease^15^ ^19–22^) or symptoms such as ‘freezing’,^23^ dysphagia^24^ or sleep disturbance.^25^ However, some studies have drawn attention to a discrepancy between patient experience and clinician understanding,^26^ ^27^ most notably at the moment of diagnosis,^2^ ^28^ ^29^ with Habermann commenting 20 years ago that it was a moment where the ‘human significance was passed over’.^29^ The intervening years have seen considerable changes to the healthcare system, including a proliferation of guidelines on how to break bad news,^iv^ a shift towards more ‘patient-centred care’,^v^ and the inclusion of communication skills training on the curricula of medical schools. And yet this study broadly concurs with the previous findings about the moment of diagnosis in the context of Parkinson's—a condition that is so much more than a ‘movement disorder’ and is now the second most common neurodegenerative disease after Alzheimer's,^30^ affecting more than 6 million people worldwide.^31^ By reassembling diagnosis conversations that are occasionally discussed but rarely heard in the literature, this paper builds on previous studies by increasing the audibility of the Parkinson's diagnosis story and recasting how it might be understood in the clinic.^32^

## Methods

### Ethics approval and recruitment methods

Ethics approval for the study was granted by the Brighton and Sussex Medical School Research Governance and Ethics Committee and the National Research Ethics Service. Sixteen women and 21 men were recruited through different routes, including the Parkinson's UK (PUK) website and local support groups, Parkinson's clinics, conferences, personal contacts and ‘snowballing’. The study aimed to be as inclusive as possible and the participants' experience of their diagnosis ranged from 3 months to 33 years, while their age at diagnosis spanned six decades, from 29 to 78. The participants came from eight different counties in the UK. All participants were given the opportunity to ask questions about information provided before completing the consent form. It was agreed that pseudonyms would be used in any reports. The participants were reminded that they could pause, take a break or simply stop the interview at any stage.

### Interviews

Face-to-face interviews, undertaken solely by the author, took the form of ‘guided conversations’ and lasted between one and one and a half hours. Rather than using a fixed set of questions, all participants were asked to talk about what it was that led them to suspect that something was ‘not quite right’, leading up to their diagnosis, and then talk about life since that moment. This approach aimed to give participants the freedom to decide for themselves how to tell their stories while nevertheless employing the same starting point. It was noticeable that participants in this study were keen to undertake and complete interviews without anyone else there, despite many suffering from challenging symptoms (eg, dyskinesia, freezing, narcolepsy or generalised discomfort) and communication difficulties. All interviews were recorded and transcribed, and listening again to interviews became as important a part of analysis as the reading and re-reading of transcripts. The stories of the diagnostic encounter emerged because participants chose to tell them, not because they were specifically questioned about it.

### Dialogical narrative analysis

The original narrative study from which diagnosis stories emerged was informed by Arthur Frank's concept of dialogical narrative analysis (DNA), a central tenet of which is to hear those stories that ‘call out as needing to be written about’.^32^ ^vi^ DNA is about the relationship between a story, a storyteller and a listener and ‘how each allows the other to be’.^34^ Its aim is not to distil participants' voices into some form of ‘truth’ nor is to end any conversation by summarising findings. Rather, it strives to ascertain how people's lives are affected by stories and allow (participants’) voices to hear one another as well as be heard collectively. Bakhtin's notion of ‘unfinalisability’ is integral to DNA since it recognises that stories have no ending because people constantly retell them in order to develop and revise their understanding of self.^32^ It is therefore an analytic approach that seemed particularly suited to a study of Parkinson's, where the trajectory of the disease is uncertain but change is inevitable and people may be constantly ‘forced’ to re-evaluate their identity. Although narrative analysis is not guided by a set of ‘formal rules’,^35^ the story is ‘taken as a whole’^36^ and my analysis, based on field note observations as well as multiple readings of transcripts,^vii^ became an iterative process of reading, writing and discussion in order to identify issues within and across transcripts.^viii^ During this process I found myself able to reassemble ‘diagnosis conversations’ from within each interview and, in the context of participants' overall narratives, diagnosis stories cried out as requiring attention, acting as both stories *behind* stories and stories *within* stories, emerging from, and integral to, the overall illness story.

In addressing the implications for clinical practice arising from accounts of diagnosis, it has been necessary to structure this paper in a way that moves away from the manner in which the broader study was reported. The participants' experiences are therefore presented in relation to three key concepts that were found to underpin the many moments of diagnosis: bareness, abandonment and the invocation of a hierarchy of neurological conditions. In the following section, the age of participants at interview and their age at diagnosis are placed in parentheses after their pseudonym and, in order to distinguish the voice of the participant from that of their consultant, any words attributed to the latter are written in bold.

## The stories

### Bareness of the diagnostic encounter

The relationship between the story, storyteller and listener was often at its most profound during participants' stories of diagnosis and strong visual images were conjured by many of their descriptions. Following ‘countless’ tests and scans, Zoe (36/29) finally saw a consultant neurologist:

…and, umm, he immediately told me that it was Parkinson's…just by looking at me…really…I obviously had that Parkinson's face…. that look. He did a few tests, there's a rigidity test and that kind of thing…but he basically just…said ‘**It's Parkinson's’** and sent me on my merry way.

Rory (48/46) also underwent months of investigation for what he understood to be a trapped nerve before seeing the neurologist who:

…very, very casually watched me walk up the corridor, down the corridor, did the tap test between index finger and thumb, tested my wrists on my left hand and right hand and said ‘**Have a seat’** and discussed the diagnosis: ‘**Well, you've got Parkinson's’**—just like that.

Time and again, participants might experience a lengthy diagnostic limbo only to be told their diagnosis *just like that*. For some, there was also the bitter–sweet memory of now being diagnosed with a condition that had initially been discounted. This was the case for Mary (52/44) who, during months of uncertainty, was told by two different doctors that she was too young to have Parkinson's. However:

When I actually got my diagnosis, it was fairly quick. I sort of tell the story, you know, [he] made me touch my nose a couple of times, and said, ‘**It's Parkinson's, off you go.**’

The apparent simplicity of these clinical tests and the brevity of the diagnostic encounter seem particularly unnerving given the life-changing nature of the diagnosis. At the time, though, she remembered thinking:

Oh good it's only Parkinson's' because ‘I'd only come across it with friends of my parents who were fairly elderly when they got it, and as far as I could see, they just shook a bit.

The inherent sense of ‘bareness’ was all the more poignant given her reflection, in hindsight, that:

I really had very little understanding of what Parkinson's was at that time.

The emotional response I experienced while interviewing participants was prompted by the descriptive bareness and the implicit lack of dignity accompanying the images described. For example, Tristan (54/49) remembered *very clearly* that:

She just you know prodded me and pushed me and sort of got me to walk and just absolutely matter of factly says “**You have Parkinson's disease”** and that's it. “**I will refer you to X to have it confirmed again—further confirmed by Y, one of the experts”,** umm, but…

For Tristan, *an unusual beast* given his long background in neuroscience research, the manner in which the diagnosis was imparted:

…probably shocked me less than it might others.’ He did, however, reflect that: ‘I think it bothered my wife, to be honest, she came along and, er, and I think she was quite upset afterwards. But, er, whether that was the information or, or the way it was imparted I'm not sure I could say. But, but it was just very business-like, simple as that.

Although he was almost certain that he would be told he had Parkinson's, it was difficult not to feel upset on his behalf that the diagnosis was so swift (he remembered being in the room for no more than 5 min), that no space was created in which he might form a reaction and that it was dealt with so routinely. Simply:

There was no great ceremony, just ‘**You have Parkinson's disease’** that's it.

It has been suggested that, in the diagnostic encounter, ‘doctor and patient sit in different positions […]—framed by diagnosis—while nonetheless sharing its impact’.^2^ This feeling of ‘shared impact’ was absent in the narratives of many participants, as revealed through the bareness of encounters that, like Tristan's, were short, business-like and lacked ‘ceremony’.

### Abandonment

#### Lack of shared impact

The absence of shared impact also fed into a sense of ‘abandonment’ that connected many accounts. This was particularly apparent in Keith's (47/29) story of his diagnosis. In his late 20s, with no inkling of what was wrong with him, he was referred to hospital by his general practitioner (GP) having lost the use of his left arm. Following 2 days of tests he recalled that:

K: “….on the Wednesday someone come through and said ‘**Well, Mr X, umm**…**we, we know what's wrong with you.’** Then another doctor comes up and they started talking between ‘em. I had to physically **ask** if they could possibly tell me what was wrong with me, what was my problem. Umm. The second doctor said ‘**Well you have Parkinson's’** and walked off. So I'm sat there, in a hospital bed…Gutted…absol…terrified really.

J: Yes.

K: Relieved to know that it was…well, not…terminal.

J: Yup. Did you know that straightaway or did you need to ask someone about that?

K: No. I had to ask someone about that. Your first thought is, sat in a wheelchair, in a corner; I'm a seventy-year-old bloke, shaking like a leaf. That is, that is every vision…It's not like that at all. Umm. The second doctor I caught hold of, he said, he said ‘**Right, we'll discharge you tomorrow.’** I said, ‘No, not until somebody's been here and explained to me exactly what the problem is.’

J: Mm.

K: So. I stopped there ‘til the following week cos I was going to have to […] and he explained to me that, umm, ‘**You have a degenerative**…**.incurable**…**lifelong disease**…**’** which is 3 things you don't really want to hear.

J: Mm.

K: I was absolutely gutted.

J: Mm.

K: I sat there and cried for 3 hours.

Similarly, for Pat (70/72), worried about a shaking hand, the lack of shared impact compounded her shock:

The GP said ‘**I don't honestly think after examining it that there's anything wrong with it, [but] I'll send you to the hospital.’** At the hospital ‘[He] made me walk up and down and then said ‘**Yes, Mrs X, you've got Parkinson's’—**literally like that. He gave me a form and said ‘**Go and have a brain scan’** and with that I was shown out of the room. I was absolutely devastated and I didn't tell the children for a month.

The distress in both Keith and Pat's stories was tangible (*I was absolutely gutted… I was devastated*) and although not stated explicitly, their way of ‘restorying’ this moment suggests that the manner of diagnosis compounded its distressing nature. For Pat, the shock of diagnosis was made all the more acute by the brevity of the encounter and a feeling of being ‘dismissed’ from the consultation room now that the final jigsaw piece had been put in place. Both her voice and body language conveyed a feeling of being abandoned as she was moved on to a different department. Keith, on the other hand, was not physically *shown out of the room* but was abandoned as he *sat there in a hospital bed* and the consultant *walked off* having delivered the news that he had Parkinson's.

#### Lack of clear guidance and systematic care

The feeling of abandonment was further perpetuated by a perceived absence of any systematic care and guidance following many participants' diagnosis. Unlike a diagnosis of cancer, where sufferers rapidly become part of a world guided by pathways, plans and support mechanisms,^ix^ the experience for people with Parkinson's is much patchier. For Keith, this resulted in a long period of denial:

Nine years I was in denial with Parkinson's…didn't want, didn't want to know about, anything about it.

Others, like Mary, left the room in which they had been diagnosed with *very little understanding* of what Parkinson's was and therefore what a diagnosis of Parkinson's might mean to them. She was, however, clear that she was *given very little information at that point.* For Sheila (53/44), there was an implicit sense that being handed some leaflets did not fit with the serious nature of the diagnosis:

I was given some leaflets to read and that, **that was it you know**, and to go back in to see him in I think it was about a month's time[….].but I didn't go back to see him for quite some time after that.

Whether the diagnosis was 2 or 20 years before, there was an explicit expectation on the part of many neurologists that patients should inform themselves about their condition. Michael (65/46), after 20 years, still recalled:

He [the doctor] said to me, ‘**I think you've got Parkinson's disease. How do you feel about that?**’ I said, ‘I've heard of it, and I don't know anything about it.’ And he said, ‘**Go and buy a book.’** And I was out of the surgery and diddlysquat, and I went and bought the book on the way out. And I got to page seven and forget it, there was this line drawing of a scrunched up man with a walking stick…I shut the book and I didn't open it again for another seven years.

For Rory, diagnosed only 2 years before I interviewed him, the difference lay only in the means by which his neurologist suggested he might inform himself:

I was just told that [‘You've got Parkinson's’] and told to come back two weeks later—‘**Google it’** and see what I made of it” adding a codicil that ‘to be fair to the neurologist… he did, he did warn me to be careful with my reading material and choice.

Perhaps not surprisingly, just as *page seven* led to Michael putting his book away for *seven years,* googling about his condition led to *some alarming moments* for Rory.

#### ‘Diagnostic silence’

The lack of guided information post-diagnosis, as well as the (perceived) ad hoc nature of follow-up appointments with a Parkinson's nurse, became all the more significant when accompanied by diagnostic silence: that is, a silence on the part of the diagnosing doctor that diagnosis ‘happens in a life that already has a story’.^37^ Not to acknowledge this was to silence the patient and ignore the degree to which a person's life story might affect, and be affected by, the way in which they received and understood their diagnosis of Parkinson's. Recounting the moment she was finally diagnosed after 7 years of unexplained symptoms, Janie (63/53) described how:

He was sort of testing me, all this business (at this point she gestured towards me with her hands outstretched, turning them at the wrist)…. cognition. And various things and he said ‘**I'll just go and see…’** I can't remember his name now, the consultant. And they were in another room, and this was bad…. The door was slightly ajar and I heard the consultant say ‘**Oh, that's Parkinson's.’** And I just sat there and thought ‘Jesus’- Sorry—because my uncle had Parkinson's. My mum's brother.

The voice she adopted as she spoke the consultant's words suggested an offhanded and dismissive tone, implying the routine nature of a diagnosis of Parkinson's. The diagnostic silence was palpable as Janie and her story remained invisible to him on the other side of the door. This silence was extended by her own doctor whose reaction to her diagnosis was “Well, you know, we'll get on to the Parkinson's nurse.” She left the room with her story untold, harbouring an underlying fear prompted by the memory of her uncle:

Well, my only thought came up—he died quite young.

She felt ‘mortified’ driving back to work and did not actually see the Parkinson's nurse until 2 months later. Instead, like Michael, she was left trying to contain her fears through the symbolic act of putting away the one book she had bought:

I went and had a coffee and flicked through it in the town and I thought, ‘Oh my God.’ All these things came up, you know, ‘I can't be doing with this.’ I actually put the book away for quite a long time…

#### ‘We will manage it, you and I’

As Janie's experience illustrates so poignantly, the complexities inherent in delivering a life-changing diagnosis mean that its insensitive handling might adversely affect how a person copes in the aftermath of their diagnosis. And yet it does not have to be like this. Following problems with her arm and shoulder, Jean (66/66) entered her consultant's room with *no idea* of the possible outcome:

[He] was very thorough, probably examined me for about three quarters of an hour, really everything, you know. My husband went with me and sat, when he'd finished he sat us down in the room and he said ‘**You've got Parkinson's’** (pause)… And I just was absolutely gobsmacked.

Understandably, as she recalled the moment of diagnosis, the shock and uncertainty were audible:

Everything went out of my head, I couldn't think what I'd got to ask him or anything, you know.

But the manner in which she storied her account revealed an unspoken understanding by Jean that her consultant acknowledged the human significance of her diagnosis.

He said, umm, ‘**It's very early stages, it's er, we will manage it, you and I, we will manage it with medication, whatever, it will not affect your life, you'll be able to carry on […] you'll be able to drive and whatever.’** He was very positive which even though I was in this gobsmacking way I thought ‘Oh, that's good you know.’

By sharing its impact (*we will manage it*), he ensured that her shock and uncertainty were ‘contained’ and that, far from feeling abandoned in her new identity, she did not leave the room facing the consequences of her diagnosis alone. Thus, in narrating her account of diagnosis, Jean was able to make a distinction between her personal reaction to the diagnosis of Parkinson's and her reaction to the way in which she was informed about it by her neurologist.

### Hierarchy of neurological conditions

No two people will react to the same diagnosis in the same way and some participants adopted a relativist position in order to make sense of their diagnosis. Richard (60/59), to whom the diagnosis of Parkinson's was both a *relief* (to know what it was) and a *bombshell,* derived some comfort from the fact that *it*'*s not cancer.* Shaped by his own life experience, he was able further to modify the *horrible spectre* of Parkinson's by reminding himself:

You know it's not a traffic accident, something like that. I mean people, people's lives are thoroughly wrecked by certain accidents or diseases. Parkinson's gets like that in the end, but you're able to plan for it.

Other participants did not necessarily choose to take this approach, but were left with little alternative by the various consultants they saw along the way, some being told that they were fortunate that it was not a brain tumour, or motor neurone disease, or Wilson's disease. Others, like Rory, received a similar message from their actual diagnosing consultant. In Rory's words, the neurologist:

…described it [Parkinson's] as probably the most benevolent of the neurological conditions he diagnoses. He said it was a very slow disease through its course, it was like a ship on the horizon and you would see it and if you watch, it's static, it's only when you turn away and come back a year later that it's moved or gone or whatever, and that was fine. He also informed me the medication was very good and very powerful, albeit that it had a finite application duration as it were, and that was fine, I understood that. And he assured me that potential cures were around the corner, the research was well-funded, well-advanced, and there's a lot of interest in science taking place. Now that was all fine…

Such assurances of *potential cures around the corner* were doubtless well intentioned, aimed at giving comfort and sustaining hope, as was the assurance that Parkinson's is one of the more ‘benevolent’ neurological conditions. However, to be told this was to be faced with a ‘hierarchical ordering of (neurological) conditions’^3^ that crowded out Rory's narrative and left him little alternative but to comment ‘that's fine’*.*

## Discussion and conclusion

The examples of the diagnostic encounter discussed in this paper emerged, not as a consequence of collecting ‘data’ about diagnosis, but as a consequence of listening carefully to the way in which study participants narrated their illness experience to a person not involved in their medical care. While they do not claim to be representative of all people diagnosed with Parkinson's, they do provide insight into the ‘generalised problem’ of the sensitivities involved in giving and receiving a diagnosis.^x^ Furthermore, these diagnosis stories emerged unencumbered by the fear that telling them might impact negatively on an individual's healthcare.

### Discussion

Narrative truth involves a ‘structured account of experience’ rather than a ‘factual record’ of what really happened,^38^ and yet the *manner* in which stories were narrated in this study is important precisely because it articulates the ‘significance and meaning of experiences’.^39^ Many participants employed direct speech as they recalled their diagnosing consultant's words, and the effect of this was powerful. It felt as though the consultant had joined the interview and it returned participants to the moment they had entered the consulting room as their ‘prior’ selves, only to leave it with their new identity of patient-with-a-diagnosis.^2^ Hearing direct speech and observing participants' body language and tone of voice further highlighted the transformative power of words. In the few seconds it takes to utter the name of a person long-dead, symptoms metamorphosed into a condition with a diagnostic classification and concomitant label—Parkinson's disease. It was the point at which the chasm between ‘lay’ and ‘expert’ knowledge was at its deepest and the new ‘patient’ was at their most vulnerable. It showed how readily the balance of power can shift in the ‘partnership’ that is at the heart of healthcare's model of patient-centred care.

The lack of a straightforward diagnostic test for Parkinson's might lead to years of diagnostic limbo^10^ or even *mis*diagnosis. Despite this, many narratives suggested that the moment of diagnosis was treated as a matter of routine by the neurologist. The final jigsaw piece had been found and, in the hierarchy of neurological diseases, things could be worse. It was not even the ‘eureka’ moment described by Pinder when concluding that diagnosis of Parkinson's was a point of ‘maximum theoretical coherence’ for the medical profession.^3^ This change might reflect the increasing specialisation in medicine over the last 25 years, and the fact that diagnoses of Parkinson's are now undertaken by specialists rather than GPs.^xi^

The existential challenges thrown up by a diagnosis of a progressive, degenerative, incurable disease remained unacknowledged at this moment. For participants, Parkinson's was seen as a label fraught with symbolic significance, steeped in the history of its former nomenclature, *the shaking palsy*,^40^ and hampered by the erroneous, yet widely held, view that Parkinson's is little more than ‘the cause of a bit of tremor in elderly folks’.^41^ It was a point at which the uncertainty and confusion of diagnostic limbo was replaced by the uncertainty and confusion of being diagnosed with a disease for which there is currently ‘finite’ treatment and, as yet, no cure, in a society where restitution remains the ‘culturally preferred narrative’^37^ and a cure remains the ultimate stated goal of scientific research.^xii^ Disappointingly, this study corroborates findings from 20 years ago where, as previously mentioned, for people with Parkinson's the ‘human significance of diagnosis was passed over’.^29^ Despite years of investment in communication skills training at medical schools, narratives revealed a real need to address the shortcomings in the way in which so many diagnoses of Parkinson's are handled.

### Conclusion

No matter who gives the diagnosis, it is surely a moment requiring ‘solicitude, empathy and support’^42^ and this article provides empirical material for discussion around how best to ensure that these qualities are present in the context of a diagnosis of Parkinson's. In order to understand what information an individual wants or is ready to hear at the moment of diagnosis, clinicians need to establish an open dialogue as well as feel confident about adapting their consultation style in response to individuals. This study suggests that the brevity of many encounters foreclosed such dialogue, as did the invocation of a hierarchy of neurological conditions. Underlying many narratives was a sense that participants felt misunderstood and abandoned, that the emotional impact of a Parkinson's diagnosis remained unacknowledged and that, in the world of neurology, Parkinson's was considered neither the ‘worst’ diagnosis nor the most ‘exciting’. In the words of one neurologist, as a profession:

We like the thrill of the chase, we love that feeling of nailing down that once elusive diagnosis. The more rare the disease, the greater the intellectual satisfaction. Eponymous syndromes, new diseases that have not yet been delineated, rare genetic disorders that the medical literature forgot….all fair game.^43^

From the biomedical view, Parkinson's might not be the worst neurological condition, but to treat its diagnosis as a matter of routine is to ignore the psycho-social aspects of a life-changing diagnosis. People might react differently to the same news, but despite individual differences all participants needed time to absorb the news of diagnosis; time to form a reaction to the news; and time to share their anxieties and fears about it. Before leaving the diagnostic consultation, participants' narratives spoke to a need to feel safe at this moment of uncertainty, back in control at a moment of disempowerment and clear that there was a plan for the future. They needed staged and guided information at the point of diagnosis rather than being told to buy a book or to ‘google’ their condition. Similarly, they did not wish to be given any sense of ‘false’ hope about their future, particularly related to possibilities of a cure. Ultimately, this study showed that there is still a need to expand the dialogue and translate the diagnosis stories shared by people with Parkinson's into a greater empathy for the disease, wherever it might lie on the neurological spectrum. Clinicians should not be left alone in undertaking this difficult task, but rather offered the benefit of a safe environment in which to share best practice and, above all, seek the views of those diagnosed with the illness.

## Footnotes

i Peter Conrad, Professor of Social Sciences, Brandeis University, paraphrasing the physician, Michael Balint, in the foreword to Annemarie Jutel's book on diagnosis, ‘Putting a Name to It; Diagnosis in Contemporary Society’ (2011).

ii The study resulted in my PhD thesis: The lived experience of Parkinson's: ‘a footprint in every room’. It formed part of the Wellcome Trust-funded London and Brighton Translation Ethics Centre project, a broad aim of which was to investigate the social impact of developments in stem cell research and neuroscience.

iii This is a quote from a consultant neurologist Romi Saha at a lunchtime talk for fellow medical professionals, given at the Princess Royal Hospital, Haywards Heath, on 31 January 2014.

iv The NICE website provides links to numerous guidelines in different areas of medicine: https://www.evidence.nhs.uk/Search?q=breaking+bad+news Accessed July 2016.

v According to a recent inquiry, “Care that is ‘patient centred’ means care that is holistic, empowering and that tailors support according to the individual's priorities and needs.” http://www.rcgp.org.uk/policy/rcgp-policy-areas/~/media/Files/Policy/A-Z-policy/RCGP-Inquiry-into-Patient-Centred-Care-in-the-21st-Century.ashx.

vi For a full discussion of DNA, see references. 32–34

vii I also wrote a number of ‘vignettes’, based on both the interview and my field note observations. This process aimed to capture the context and overall feeling of interviews, taking into account how participants presented themselves on that particular day, as well as my role as an interviewer. It proved a valuable starting point for gaining a sense of the narrative structure of an interview and attuning me to general themes emerging from individual stories.

viii I was fortunate to have a medical sociologist and an ethicist as my supervisors with whom I was able to discuss transcripts.

ix http://www.nhsimas.nhs.uk/ist/delivering-cancer-wait-times/ (accessed Jul 2016).

x Although this is a qualitative study, it is noteworthy that the bareness of the diagnostic encounter was explicit in at least 2/3 of the participants’ interviews, irrespective of gender, age at diagnosis or length of time since diagnosis.

xi In the UK: NICE guidelines since 2006 have specified that ‘people with suspected PD should be referred quickly (ie, within 6 weeks) and untreated to a specialist with expertise in the differential diagnosis of this condition’ (p. 6).

xii This goal is reflected in the work of large national charities such as PUK, whose strapline is: ‘Change Attitudes. Find a Cure. Join Us’ (2016).
